# Non-alcoholic fatty liver disease in patients with morbid obesity: the gut microbiota axis as a potential pathophysiology mechanism

**DOI:** 10.1007/s00535-023-02075-7

**Published:** 2024-01-24

**Authors:** Isabel Cornejo-Pareja, Mohamed Reda Amiar, Luís Ocaña-Wilhelmi, Rocío Soler-Humanes, Isabel Arranz-Salas, Lourdes Garrido-Sánchez, Carolina Gutiérrez-Repiso, Francisco Jose Tinahones

**Affiliations:** 1 Department of Endocrinology and Nutrition, Virgen de la Victoria University Hospital, Malaga University, Campus Teatinos S/N, 29010 Málaga, Spain; 2 Instituto de Investigación Biomédica de Málaga-Plataforma BIONAND (IBIMA), Virgen de la Victoria University Hospital, Malaga University, 2ª Planta, Campus Teatinos S/N, 29010 Málaga, Spain; 3 Centro de Investigacion Biomedica en Red de la Fisiopatología de la Obesidad y Nutricion (CIBEROBN), Instituto de Salud Carlos III (ISCIII), 29010 Málaga, Spain; 4 Department of Medicine and Dermatology, Faculty of Medicine, University of Málaga, 29010 Málaga, Spain; 5 Department of Clinical Analysis Laboratory, Virgen de la Victoria Hospital, 29010 Málaga, Spain; 6 Department of General and Digestive Surgery, Virgen de la Victoria University Hospital, 29010 Málaga, Spain; 7 Department of Surgical Specialities, Biochemistry and Immunology, Faculty of Medicine, University of Málaga, 29010 Málaga, Spain; 8 Department of Human Physiology, Human Histology, Anatomical Pathology and Physical Education, Malaga University, 29010 Málaga, Spain; 9 Department of Anatomical Pathology, Virgen de la Victoria Hospital, Málaga, Spain

**Keywords:** Metabolic-associated fatty liver disease (MAFLD), Non-alcoholic steatohepatitis (NASH), Hepatic steatosis, Gut microbiota pattern composition and functionality, Morbid obesity

## Abstract

**Background/aim:**

Alterations in gut microbiota are associated with the pathogenesis of metabolic diseases, including metabolic-associated fatty liver disease (MAFLD). The aim of this study was to evaluate gut microbiota composition and functionality in patients with morbid obesity with different degrees of MAFLD, as assessed by biopsy.

**Subjects/methods:**

110 patients with morbid obesity were evaluated by biopsy obtained during bariatric surgery for MAFLD. Stool samples were collected prior to surgery for microbiota analysis.

**Results:**

Gut microbiota from patients with steatosis and non-alcoholic steatohepatitis (NASH) were characterized by an enrichment in *Enterobacteriaceae* (an ethanol-producing bacteria), *Acidaminococcus* and *Megasphaera* and the depletion of *Eggerthellaceae* and *Ruminococcaceae* (SCFA-producing bacteria). MAFLD was also associated with enrichment of pathways related to proteinogenic amino acid degradation, succinate production, menaquinol-7 (K2-vitamin) biosynthesis, and saccharolytic and proteolytic fermentation. Basic histological hepatic alterations (steatosis, necroinflammatory activity, or fibrosis) were associated with specific changes in microbiota patterns. Overall, the core microbiome related to basic histological alterations in MAFLD showed an increase in *Enterobacteriaceae* and a decrease in *Ruminococcaceae*. Specifically, *Escherichia coli* was associated with steatosis and necroinflammatory activity, whilst *Escherichia-shigella* was associated with fibrosis and necroinflammatory activity.

**Conclusions:**

We established a link between gut microbiota alterations and histological injury in liver diagnosis using biopsy. Harmful products such as ethanol or succinate may be involved in the pathogenesis and progression of MAFLD. Thus, these alterations in gut microbiota patterns and their possible metabolic pathways could add information to the classical predictors of MAFLD severity and suggest novel metabolic targets.

**Supplementary Information:**

The online version contains supplementary material available at 10.1007/s00535-023-02075-7.

## Introduction

Obesity is a complex multifactorial disease that is considered a state of chronic low-grade inflammation and is linked to an increased risk of many complications and an overall increase in mortality. Non-alcoholic fatty liver disease (NAFLD) is closely associated with obesity and has a reported prevalence of up to 80% in this population [1].

NAFLD is a common cause of chronic liver disease characterized by excessive fat deposition in the liver without secondary origins. In obesity, the capacity of adipose tissue to store excess energy is overwhelmed; thus, fat is redistributed to ectopic stores such as liver tissue. In this situation, hepatocytes have been attributed to an adipocyte-like function [2]. NAFLD encompasses a broad spectrum of liver disease presentations that can progress from simple steatosis to non-alcoholic steatohepatitis (NASH), and even end-stage liver disease or cirrhosis [3].

There is an ongoing debate whether NAFLD reflects the current understanding of liver disease, and metabolic-associated fatty liver disease (MAFLD) has been suggested as an appropriate term that more accurately describes hepatic manifestation of systemic metabolic dysfunction [4].

The pathophysiology of MAFLD and its progression are induced by the interaction of multiple factors, such as genetic factors (some specific polymorphisms), environmental influences (unhealthy diet, lack of physical activity), epigenetic modifications, endocrine disruptors, obesity (insulin resistance, adipokine dysregulation), lipotoxicity, endoplasmic reticulum stress, oxidative stress, and gut microbiota dysbiosis [5].

The gut microbiota is considered to be a virtual metabolic organ involved in the pathogenesis of numerous metabolic diseases [6]—obesity, dyslipidemia, type 2 diabetes mellitus, atherosclerosis, and MAFLD. Alterations in gut microbiota composition and/or its functionality might contribute to disturbed energy and substrate metabolism, including effects on metabolism and liver injury. In particular, changes in the microbial composition involved in the conversion of bile acids, the production of short chain fatty acids (SCFA), the promotion of chronic exposure to pathogen-associated molecular patterns such as lipopolysaccharides (LPS), peptidoglycans, or trimethylamine (TMA), and oxidative stress caused by increased endogenous ethanol or those involved in immune regulation and IgA production [7, 8].

In recent years, different studies have investigated the composition of gut microbiota in MAFLD. Taken together, these studies describe a different composition of gut microbiota related to the presence of MAFLD. A recent meta-analysis [9] exposed specific bacterial changes, with increased abundance of *Escherichia, Prevotella, Streptococcus* and decreased abundance of *Coprococcus, Faecalibacterium and Ruminococcus*, as gut microbiota signatures of MAFLD. The discrepancies between studies may be associated with geographic origins or dietary habitat [10], but partway due to the different techniques that may have been used in the diagnosis of MAFLD.

MAFLD can be identified using non-invasive tools such as blood-based biomarkers and liver scores [11] or proton magnetic resonance spectroscopy, computed tomographic scanning, ultrasonography, or transient elastography (fibroscan) [12, 13]. However, since non-invasive procedures show less accuracy in discriminating simple steatosis from NASH, liver biopsy is considered the gold standard assessment for the diagnosis of MAFLD [12].

The aim of this study is to describe the composition and predicted functionality of gut microbiota in patients with morbid obesity undergoing bariatric surgery with different degrees of MAFLD assessed by liver biopsy. Second, we analyzed gut microbiota composition according to different histological alterations.

## Methods

### Setting study and subject

This single-centre, transversal cohort study was approved by and in accordance with the recommendations of the Biomedical Research Ethics Coordinator Committee of Andalucía (CCEIBA), and all patients provided written consent confirming their willingness to participate in the study.

Between 2018 and 2021, 110 patients with morbid obesity who were consecutively included in the surgical waiting list for bariatric surgery at Virgen de la Victoria University Hospital were invited to participate in this study. The inclusion criteria were the acceptance of informed consent for liver biopsies to be performed during bariatric surgery and the provision of a stool sample for microbiota analysis prior to surgery.

Patients were excluded if they had cardiovascular, acute inflammatory, or infectious diseases. Patients receiving ursodeoxycholic acid treatment were also excluded. The use of antibiotics, probiotics, or prebiotic supplements that could modify microbiota in the previous 3 months was grounds for exclusion.

### Anthropometric and laboratory measurements

Before surgery, anthropometric measurements were recorded according to standardized procedures and body mass index (BMI) was calculated as weight (kg)/height^2^ (m^2^). Blood samples were collected after 12-h fast and serum was separated and immediately frozen at − 80 °C. Faecal samples were collected before bariatric surgery and immediately frozen at − 80 °C until analysis.

Serum biochemical parameters, including glucose, triglycerides, total cholesterol, and high-density lipoprotein cholesterol (HDL-c), were analyzed using an Advia Chemistry XPT autoanalyzer (Siemens Healthcare Diagnostics). The coefficients of variation for glucose, triglyceride, total cholesterol, and HDL-c were 1.8%, 2.5%, 3.9%, and 4.5%, respectively. Low-density lipoprotein cholesterol (LDL-c) levels were calculated using Friedewald’s formula [14]. Serum insulin levels were measured using an immunoassay (ADVIA Centaur Autoanalyzer, Siemens Healthcare Diagnostics). Homeostasis model assessment of insulin resistance (HOMA-IR) was calculated using the formula: fasting insulin (μIU/mL) × fasting glucose (mmol/L)/22.5 [15].

### Histological analysis

Histological analysis of the liver biopsies was performed based on the Brunt semi-quantitative classification [16]. Liver steatosis is an infiltration of hepatic fat with minimal inflammation and is graded based on the fat percentage in hepatocytes: grade 0 (< 5%), grade 1 (5%–33%), grade 2 (33%–66%), and grade 3 (> 66%) [17].

Necroinflammatory activity is manifested by two factors: the inflammatory lobular: no inflammation (grade 0), scattered neutrophils with or without lymphocytes (< 2 groups) (grade 1), intralobular neutrophils (2–4 groups) (grade 2), marked lobular inflammation (> 4 groups neutrophils) (grade 3), and the presence of hepatocyte ballooning degeneration: no ballooned cells (grade 0), few ballooned cells (grade 1), and many ballooned cells (grade 2) [17].

Liver fibrosis was graded based on the increase in connective tissue deposition and architectural remodelling noted: perisinusoidal or pericellular fibrosis (stage 1), perisinusoidal or pericellular fibrosis with focal or extensive periportal fibrosis (stage 2), perisinusoidal/pericellular fibrosis and portal fibrosis with focal or extensive bridging fibrosis (stage 3), and cirrhosis, progression of collagen deposits to severe fibrosis: pericellular, portal, and extensive bridging fibrosis (stage 4) [17].

Patients were classified according to the histological study into three groups: control group (patients with morbid obesity and non-fibrosis, non-steatosis, and non-necroinflammatory activity), patients with morbid obesity plus steatosis grade ≥ 1 (non-fibrosis and non-necroinflammatory activity), and patients with morbid obesity and steatosis grade ≥ 1 plus NASH (necroinflammatory activity grade ≥ 1 with/without fibrosis).

In addition, patients were separated according to the presence or absence of individual basic histological features: steatosis (grade ≥ 1) vs. non-steatosis (grade = 0), necroinflammatory activity (grade ≥ 1) vs. non-necroinflammatory activity (grade = 0), and fibrosis (stage ≥ 1) vs. non-fibrosis (stage = 0).

### Analysis of gut microbiota

DNA was extracted from stool samples using a QIAamp DNA Stool Mini Kit (QIAGEN Science, Hilden, Germany), following the manufacturer’s instructions. DNA concentration and purity were determined using a NanoDrop spectrophotometer (NanoDrop Technologies Inc., Wilmington, DE, USA). Libraries were built using the Ion 16S Metagenomics kit and Ion Plus Fragment Library kit (Thermo-Fisher Scientific Inc., Waltham, MA, USA) as previously described [18]. Sequencing of the amplicon libraries was carried out on an Ion 520 chip using Ion Torrent S5 (Thermo-Fisher Scientific Inc., Waltham, MA, USA) according to the manufacturer’s instructions.

### Sequence data analysis

Base calling and run demultiplexing were performed using Torrent Suite TM Server software (Thermo-Fisher Scientific Inc., Waltham, MA, USA), version 5.4.0, with default parameters for 16S Target Sequencing (bead loading ≤ 30, key signal ≤ 30, and usable sequences ≤ 30). Quality sequences were analyzed using QIIME2 2022.2 software (Quantitative Insights into Microbial Ecology). Unique amplicon sequence variants (ASVs) were calculated using DADA2 [19].

Taxonomic classification of features was based on SILVA version 138, 99% clustering similarity database. The SILVA reference sequence and taxonomy were obtained as pre-formatted files (https://docs.qiime2.org/2022.2/data-resources/) that were processed using RESCRIPt plugin from QIIME 2. ASVs classified as chloroplasts or mitochondria were excluded from downstream analysis. Features with a count sum of less than 10 across all samples and those presented in only one sample were removed.

Diversity analysis was performed using the core-metrics-phylogenetic plugin in QIIME2, after randomly subsampling the samples to obtain the same number of sequences. Statistical differences in alpha diversity metrics were calculated using the Kruskal–Wallis test using Benjamini–Hochberg correction for multiple comparisons. Statistical differences in beta diversity distances were assessed using PERMANOVA test with Benjamini–Hochberg correction for multiple comparisons. Amplicon sequence variant tables were analyzed at different taxa levels using linear discriminant analysis (LDA) effect size method (LEfSe) to test differences in abundance between groups within MicrobiomeAnalyst webtool with the default parameters of the developer (LDA > 2; *p* < 0.05) [20].

The predicted functional profiles of the microbial communities were calculated using Phylogenetic Investigation of Communities by Reconstruction of Unobserved States (PICRUSt2) plugin in QIIME2. Metacyc pathways were analyzed using STAMP software (Statistical Analysis of Metagenomic Profiles) [21].

### Statistical analysis

To summarize the anthropometric and biochemical characteristics of the cohort, a descriptive analysis was performed using measurements of central tendency and dispersion (mean ± standard deviation (SD)). For comparison of continuous variables, Mann–Whitney test was used. Between-group comparisons of categorical variables were performed using the *χ*^2^ test. The significance level was set at *p* < 0.05. Results are presented as mean ± standard deviation (SD).

## Results

### Anthropometric and biochemical characteristics of the patients included in the study

We evaluated a sample of 110 patients; 70% were female with a mean age of 46.76 + 8.91 years. No significant differences were found between the subgroups. The main anthropometric and biochemical characteristics of the patients are shown in Table 1. Age, glucose levels, insulin levels, HOMA-IR values, cholesterol, and triglyceride levels were significantly higher in steatosis plus NASH group than in the control group. There were no statistically significant differences between groups in the other variables that evaluated hydrocarbon and lipid metabolism or liver enzymes. No statistically significant differences were found between control group and steatosis group.

**Table 1:** Anthropometric and biochemical variables

	Control	Steatosis	NASH + steatosis
Sex (F/M)	24/12	15/5	38/16
Age (years)	44.31 ± 9.51	46.30 ± 8.09	48.57 ± 8.51*
Weight (kg)	132.95 ± 20.23	130.32 ± 20.42	136.43 ± 24.26
BMI (kg/m^2^)	46.95 ± 6.18	47.26 ± 6.97	49.67 ± 6.71
Glucose (mg/dl)	103.17 ± 22.50	103.95 ± 13.99	116.79 ± 33.46*
Insulin (µUI/ml)	14.35 ± 7.35	19.05 ± 10.38	26.21 ± 32.22*
HOMA-IR	3.72 ± 2.01	4.89 ± 2.59	7.48 ± 8.00*
Cholesterol (mg/dl)	181.57 ± 35.48	190.63 ± 40.70	195.53 ± 42.62*
Triglycerides (mg/dl)	124.14 ± 64.35	161.63 ± 46.51	183.02 ± 106.42*
HDL-cholesterol (mg/dl)	44.03 ± 13.45	45.32 ± 13.73	45.08 ± 11.82
LDL-cholesterol (mg/dl)	113.74 ± 30.96	119.00 ± 29.87	115.44 ± 33.71
GGT (U/l)	30.54 ± 21.71	28.79 ± 17.75	37.91 ± 30.09
AST (U/l)	24.06 ± 9.30	25.89 ± 11.07	30.14 ± 13.28
ALT (U/l)	29.09 ± 13.04	28.79 ± 17.75	35.13 ± 16.42
SBP (mm Hg)	134.56 ± 25.18	127.25 ± 16.03	132.75 ± 16.58
DBP (mm Hg)	84.58 ± 14.53	81.35 ± 8.55	84.42 ± 10.75
Histological parameters (*n*)			
Steatosis grade score (0/1/2/3)	36/0/0/0	0/17/0/3	0/31/14/9
Necroinflammatory activity grade (0/1/2/3)	36/0/0/0	20/0/0/0	0/38/16/0
Fibrosis state (0/1/2/3/4)	36/0/0/0/0	20/0/0/0	18/22/9/5

*BMI* Body mass index, *GGT* gamma-glutamyl transferase, *AST* aspartate aminotransferase, *ALT* alanine aminotransferase, *HOMA-IR* homeostasis model assessment of insulin resistance, *SBP* systolic blood pressure, *DBP* diastolic blood pressure

*Kruskal–Wallis test adjusted by Bonferroni, *p* < 0.05, between control group and steatosis + NASH group

*p < 0.05 between control group and NASH + steatosis group using Mann–Whitney *U* test

### Gut microbiota diversity and MAFLD progression

**Fig. 1:**
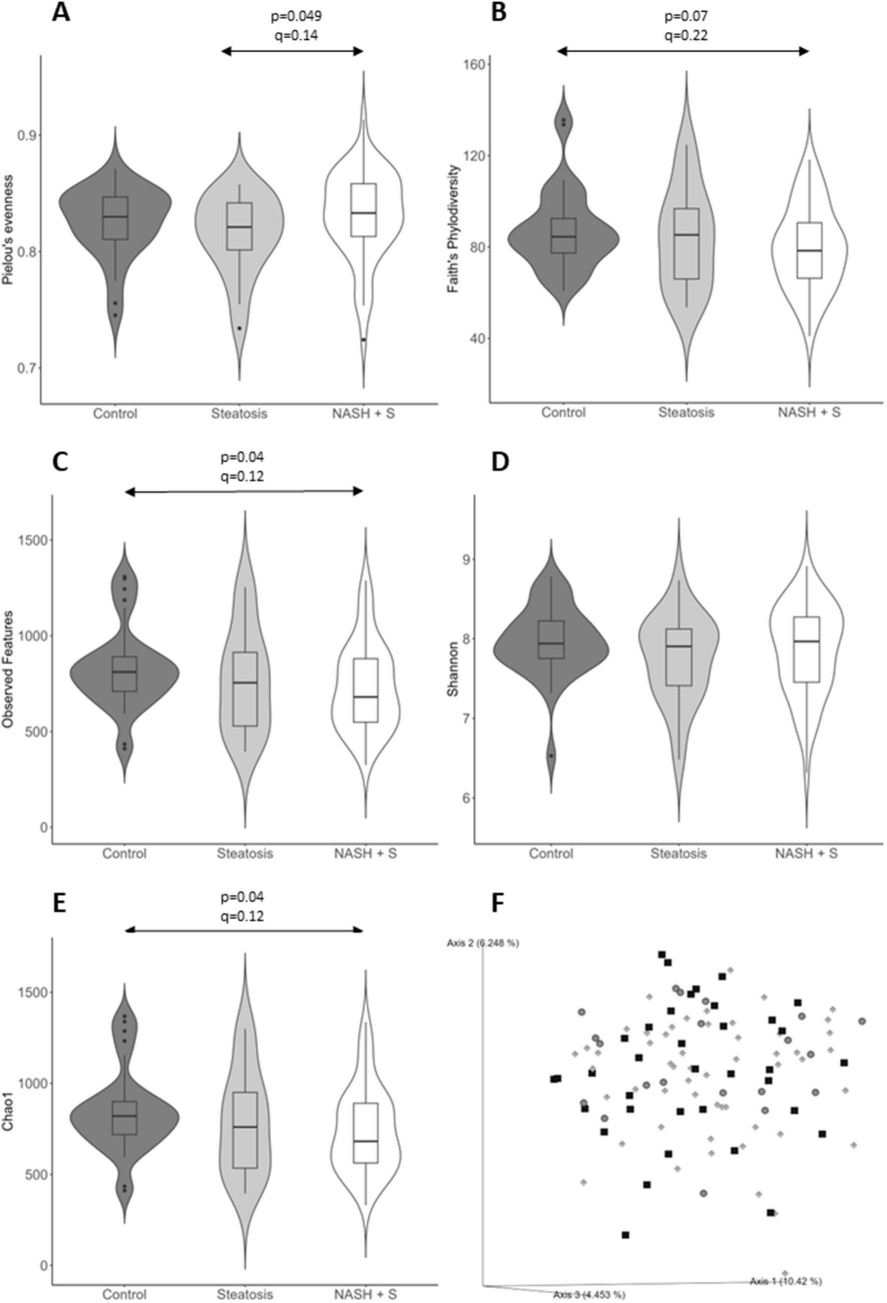
Gut microbiota diversity. **A** Pielou’s evenness. **B** Faith’s phylodiversity. **C** Observed features. **D** Shannon. **E** Chao1. **F** Principal coordinates analysis plot of the unweighted UniFrac distance. Black squares: control group. Grey circles: steatosis group. Light grey diamonds: steatosis + NASH group

Alpha-diversity analysis showed an increase in Pielou’s evenness in steatosis plus NASH group compared to steatosis group (*p* = 0.049), whilst Observed Features index and Chao-1 index were significantly higher in control group compared to steatosis plus NASH group (*p* = 0.04 in both cases). However, after correction for multiple comparisons, differences were not significant (*q* = 0.14, *q* = 0.12 and *q* = 0.12, respectively). Faith’s Phylodiversity index showed a tendency to be higher in control group compared to steatosis plus NASH group (Fig. 1). In contrast, beta-diversity analysis showed that Unweighted UniFrac distance was significantly different between steatosis plus NASH and control group (PERMANOVA, pseudo-F: 1.16, *p* = 0.032, *q* = 0.096) (Fig. 1).

### Gut microbiota composition and its predicted functional properties in MAFLD progression

**Fig. 2:**
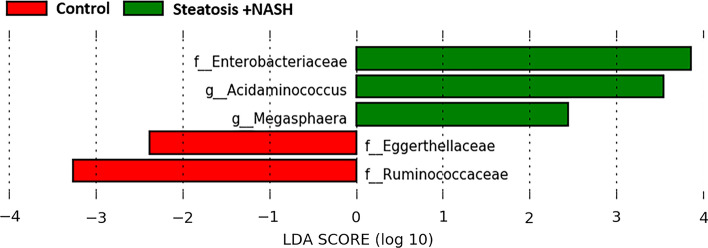
Significantly different taxa identified by linear discriminant analysis effect size (LEFSe) in hepatic disease (LDA score > 2 *p* < 0.05)

Linear discriminant analysis effect size (LEfSe) showed that steatosis plus NASH was enriched in *Enterobacteriaceae* family and *Acidaminococcus* and *Megasphaera* genera, whereas control group was enriched in *Eggerthellaceae* and *Ruminococcaceae* families (Fig. 2). However, no significant enrichment was observed in steatosis group.

**Fig. 3:**
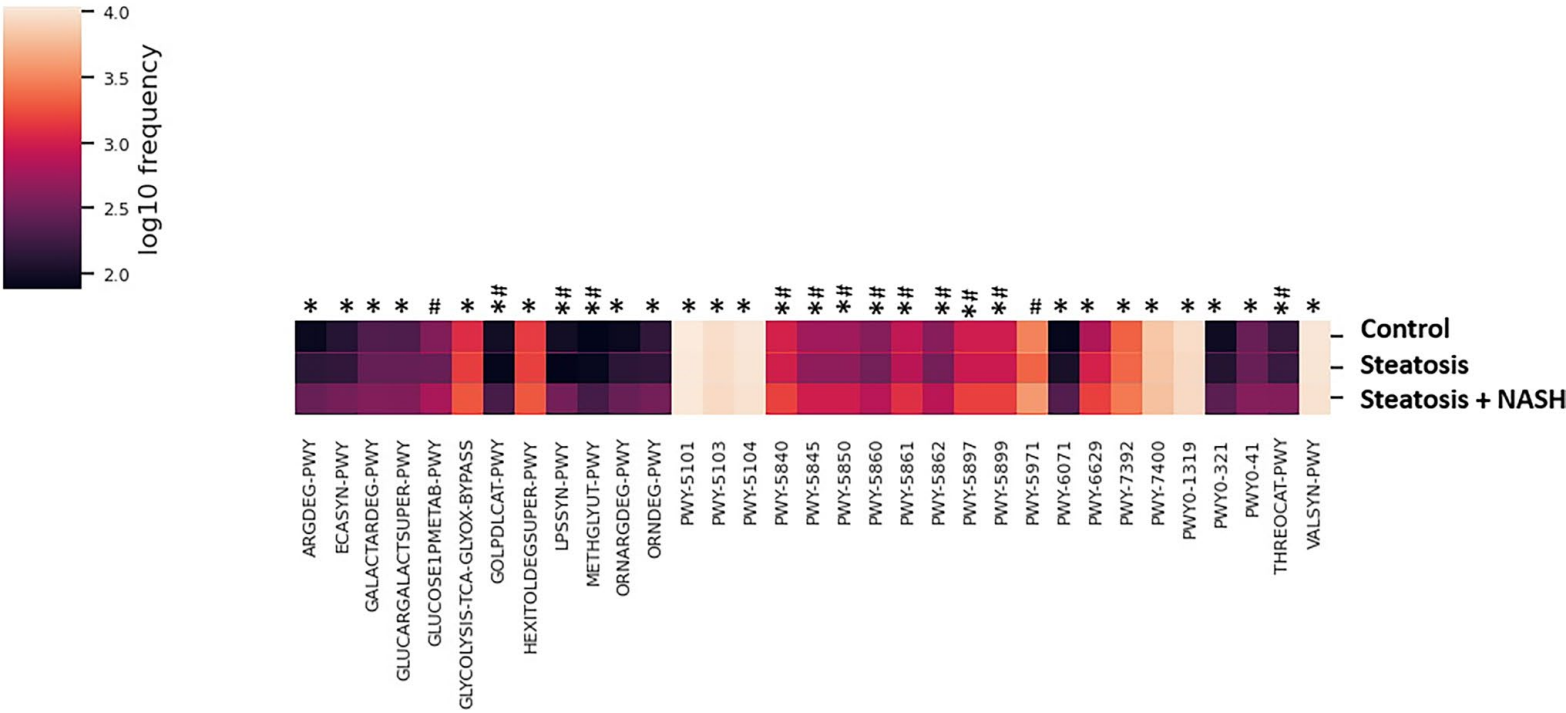
Heatmap representing the significant predictive metabolic pathways by PICRUSt2. Kruskal–Wallis non-parametric test with Benjamini–Hochberg FDR (false discovery rate) < 0.1. *FDR < 0.1 between steatosis plus NASH and control group. #FDR < 0.1 between steatosis plus NASH and Steatosis group

Predicted functional analysis based on PICRUSt2 revealed that steatosis plus NASH group showed an enrichment in pathways related to proteinogenic amino acid degradation, such as l-arginine and l-threonine degradation (ARGDEG-PWY, ORNARGDEG-PWY, THREOCAT-PWY), but also an enrichment in proteinogenic amino acid biosynthesis, such as l-isoleucine, l-tryptophan, and l-arginine biosynthesis (PWY-5101, PWY-5103, PWY-5104, PWY-6629 PWY-74100, and VALSYN-PWY compared to control group, as well as an enrichment in the biosynthesis of quinol and quinone (PWY-5840, PWY-5845, PWY-5850, PWY-5860, PWY-5861, PWY5862, PWY-5897, and PWY-5899) and the degradation of sugar acid (GALACTARDEG-PWY and GLUCARGALACTSUPER-PWY) and amine and polyamine (ORNDEG-PWY and PWY-6071) was observed in steatosis plus NASH group compared to control group (Fig. 3).

The aforementioned enrichment in the quinol and quinone biosynthesis pathways was also observed in steatosis plus NASH group compared to steatosis group (Fig. 3).

### Gut microbiota and MAFLD alterations

To analyze to what extent simple histological hepatic alterations, such as fibrosis, steatosis, and necroinflammatory activity, were associated with changes in gut microbiota, patients were classified according to the presence/absence of these alterations in liver biopsy. Table 2 shows the differences between groups according to the classification of basic histological characteristics (fibrosis, steatosis, or necroinflammatory activity). Patients with necroinflammatory activity or steatosis were significantly older than those without necroinflammatory activity or steatosis, respectively. In addition, patients with any type of morphological liver injury (fibrosis, steatosis, or necroinflammatory activity) had significantly altered hydrocarbon metabolism, mainly insulin resistance, as well as significantly higher triglyceride levels. We detected an increase in liver enzyme levels only in those patients with liver fibrosis.

**Table 2:** Anthropometric and biochemical variables according to the classification criteria

	Non-necrosis	Necrosis
Sex (F/M)	39/17	38/16
Age (years)	45.02 ± 9.01	48.57 ± 8.51*
Weight (kg)	132.01 ± 20.15	136.43 ± 24.26
BMI (kg/m^2^)	47.06 ± 6.41	49.67 ± 6.71*
Glucose (mg/dl)	103.44 ± 19.84	116.79 ± 33.46*
Insulin (µUI/ml)	15.97 ± 8.72	26.21 ± 32.22*
HOMA-IR	4.12 ± 2.27	7.48 ± 8.00*
Cholesterol (mg/dl)	184.76 ± 37.27	195.53 ± 42.62
Triglycerides (mg/dl)	126.78 ± 58.34	183.02 ± 106.42*
HDL-cholesterol (mg/dl)	44.48 ± 13.44	45.08 ± 11.82
LDL-cholesterol (mg/dl)	115.59 ± 30.41	115.44 ± 33.71
GGT (U/l)	29.93 ± 20.25	37.91 ± 30.09
AST (U/l)	24.67 ± 9.75	30.14 ± 13.28
ALT (U/l)	30.57 ± 14.83	35.13 ± 16.42
SBP (mm Hg)	131.95 ± 22.47	132.75 ± 16.58
DBP (mm Hg)	83.43 ± 12.73	84.42 ± 10.75
Histological parameters (*n*)		
Steatosis grade score (0/1/2/3)	36/17/0/3	0/31/14/9
Necroinflammatory activity grade (0/1/2/3)	56/0/0/0	0/38/16/0
Fibrosis state (0/1/2/3/4)	56/0/0/0	18/19/11/6

	Non-steatosis	Steatosis
Sex (F/M)	24/12	53/21
Age (years)	44.31 ± 9.51	47.96 ± 8.41*
Weight (kg)	132.95 ± 20.23	134.78 ± 23.31
BMI (kg/m^2^)	46.95 ± 6.18	49.02 ± 6.82
Glucose (mg/dl)	103.17 ± 22.50	113.40 ± 30.04
Insulin (µUI/ml)	14.35 ± 7.35	24.32 ± 28.24*
HOMA-IR	3.72 ± 2.01	6.80 ± 7.06*
Cholesterol (mg/dl)	181.57 ± 35.48	194.24 ± 41.89
Triglycerides (mg/dl)	124.14 ± 64.35	169.46 ± 96.76*
HDL-cholesterol (mg/dl)	44.03 ± 13.45	45.14 ± 12.25
LDL-cholesterol (mg/dl)	113.74 ± 30.96	116.39 ± 32.55
GGT (U/l)	30.54 ± 21.71	35.50 ± 27.56
AST (U/l)	24.06 ± 9.30	29.11 ± 12.77
ALT (U/l)	29.09 ± 13.04	34.65 ± 16.67
SBP (mm Hg)	134.56 ± 25.18	131.25 ± 16.50
DBP (mm Hg)	84.58 ± 14.53	83.58 ± 10.23
Histological parameters (*n*)		
Steatosis grade score (0/1/2/3)	36/0/0/0	0/48/14/12
Necroinflammatory activity grade (0/1/2/3)	36/0/0/0	20/38/16/0
Fibrosis state (0/1/2/3/4)	36/0/0/0	38/19/11/6

	Non-fibrosis	Fibrosis
Sex (F/M)	52/22	25/11
Age (years)	45.99 ± 8.84	48.36 ± 8.96
Weight (kg)	133.43 ± 19.87	135.71 ± 26.79
BMI (kg/m^2^)	47.87 ± 6.57	49.31 ± 6.83
Glucose (mg/dl)	105.68 ± 22.26	118.61 ± 35.89
Insulin (µUI/ml)	19.69 ± 27.51	23.61 ± 13.96*
HOMA-IR	5.08 ± 6.25	7.16 ± 5.42*
Cholesterol (mg/dl)	185.08 ± 39.89	199.97 ± 39.45
Triglycerides (mg/dl)	136.70 ± 68.99	190.00 ± 113.76*
HDL-cholesterol (mg/dl)	44.59 ± 12.44	45.14 ± 13.10
LDL-cholesterol (mg/dl)	113.66 ± 32.07	119.28 ± 31.72
GGT (U/l)	30.46 ± 20.94	40.61 ± 32.67*
AST (U/l)	24.06 ± 9.17	32.17 ± 13.75*
ALT (U/l)	30.01 ± 14.00	38.39 ± 17.60*
SBP (mm Hg)	131.56 ± 20.55	133.92 ± 18.14
DBP (mm Hg)	84.70 ± 12.39	82.31 ± 10.37
Histological parameters (*n*)		
Steatosis grade score (0/1/2/3)		
Necroinflammatory activity grade (0/1/2/3)		
Fibrosis state (0/1/2/3/4)		

*BMI* Body mass index, *GGT* Gamma-glutamyl transferase, *AST* aspartate aminotransferase, *ALT* alanine aminotransferase, *HOMA-IR *homeostasis model assessment of insulin resistance, *GGT* gamma-glutamyl transferase, *SBP* systolic blood pressure, *DBP* diastolic blood pressure

Mann–Whitney test, *p < 0.05 between groups using Mann–Whitney test

Diversity analysis showed no significant differences in α-diversity indexes (Supplementary Fig. 1), regarding to β-diversity unweighted UniFrac distance measure showed differences according to the presence of fibrosis (PERMANOVA, pseudo-F: 1.54, *p* = 0.02) and necroinflammatory activity (PERMANOVA, pseudo-F: 1.40, *p* = 0.049).

**Fig. 4:**
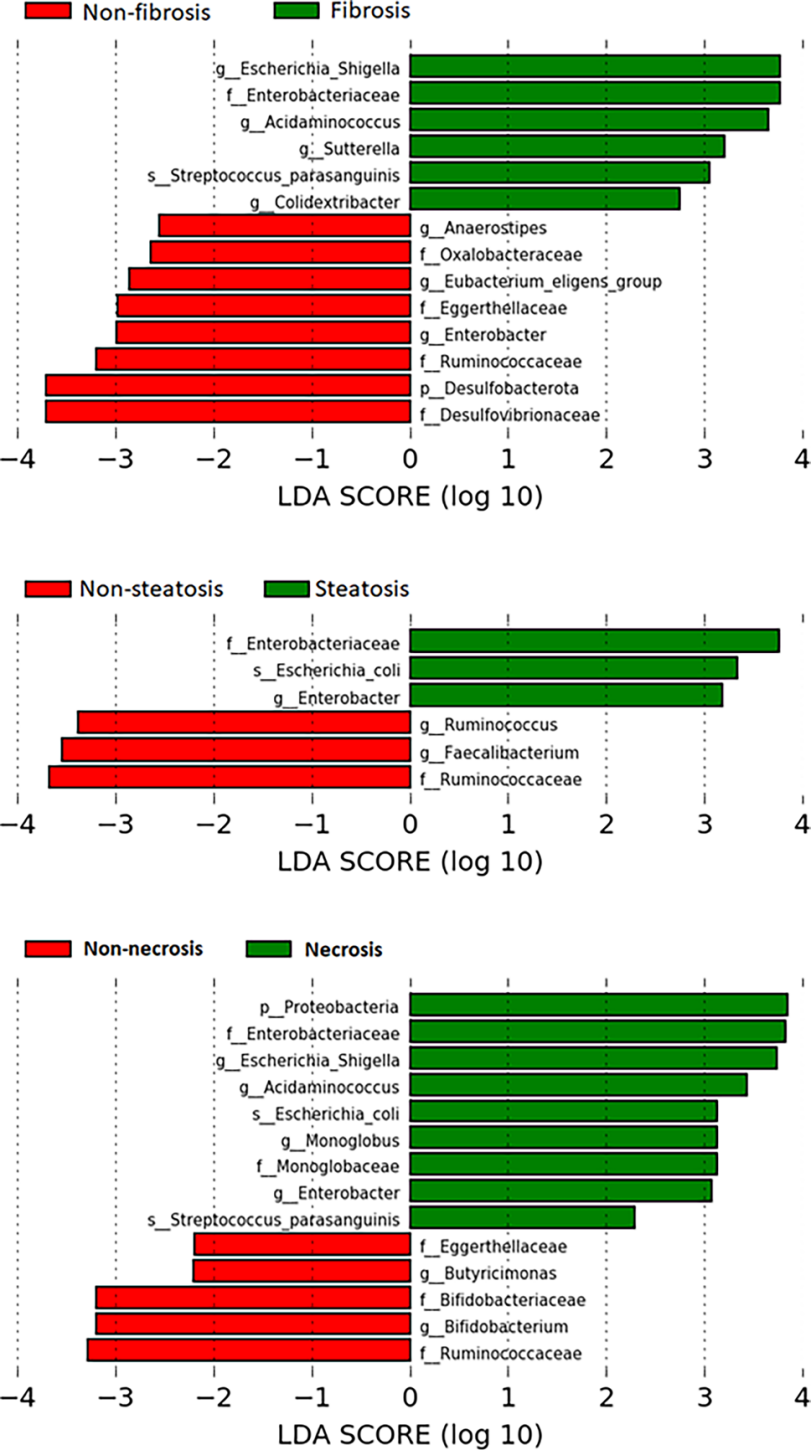
Significantly different taxa identified by linear discriminant analysis effect size (LEFSe) in hepatic alterations (LDA score > 2 *p* < 0.05)

Fibrosis was shown to be associated with an enrichment in *Enterobacteriaceae* family and *Escherichia_Shigella, Acidaminococcus, Sutterella* and *Colidextribacter* genera, while steatosis was associated with an enrichment in *Enterobacteriaceae* family and *Enterobacter* genus. Moreover, an enrichment in *Enterobacteriaceae* and *Monoglobaceae* families and *Escherichia_Shigella*, *Acidaminococcus*, *Monoglobus* and *Enterobacter* genera was associated with the presence of necroinflammatory activity (Fig. 4).

*Enterobacteriaceae* was shown to be enriched in fibrosis, as well as steatosis and necroinflammatory status, whilst *Escherichia_Shigella, Acidaminococcus* and *Streptococcus parasanguinis* were enriched in both fibrosis and necroinflammatory activity. Moreover, *Enterobacter* and *Escherichia coli* were enriched in steatosis and necroinflammatory activity (Supplementary Fig. 2).

In Supplementary Fig. 3 are shown the relative abundance of the most representative altered taxa according to the grade of pathological feature liver disease.

**Fig. 5:**
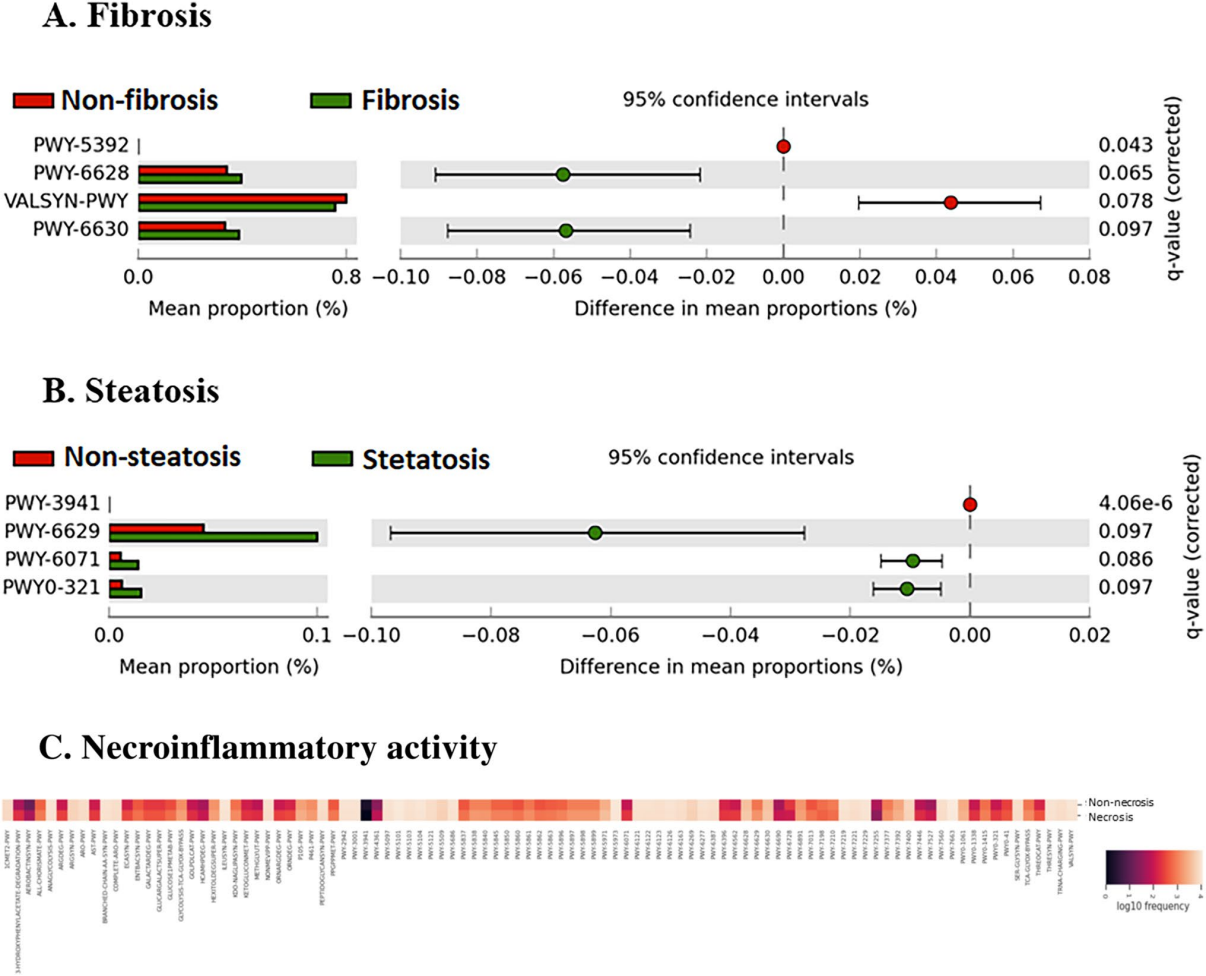
Predictive metabolic pathways by PICRUSt2 in fibrosis (**A**) steatosis (**B**) and necroinflammatory activity (**C**). White’s non-parametric test with Benjamini–Hochberg FDR (false discovery rate) < 0.1

Regarding to predicted functionality based on PICRUSt2, fibrosis was distinguished by an enrichment in the biosynthesis of proteinogenic amino acids such as l-phenylalanine and l-tyrosine (PWY-6628, PWY-6630) (Fig. 5A), whilst steatosis showed an enrichment in the degradation of phenolic compounds (PWY-6071, PWY0-321) and the biosynthesis of l-tryptophan (PWY-6629) (Fig. 5B). Moreover, necroinflammatory activity showed the highest number of altered metabolic pathways, with an enrichment in pathways related to the degradation of aromatic compounds (3-HYDROXYPHENYLACETATE-DEGRADATION-PWY, HCAMHPDEG-PWY, PWY-6690, PWY0-321), amine and polyamine (PWY0-41, ORNDEG-PWY, PWY-6071), and l-arginine and l-threonine proteinogenic amino acids (ARGDEG-PWY, AST-PWY, ORNARGDEG-PWY, THREOCAT-PWY), although an enrichment in the biosynthesis of other proteinogenic amino acids, such as l-phenylalanine, l-tryptophan, l-tyrosine, l-methionine, and l-alanine, was predicted (PWY-6628, PWY-6629, PWY-6630, PWY-7527, PWY0-1061). In addition, an enrichment in the biosynthesis of quinol and quinones was predicted in patients with necroinflammatory activity (PWY-5838, PWY-5840, PWY-5845, PWY-5850, PWY-5860, PWY-5861, PWY-5862, PWY-5863, PWY-5896, PWY-5897, PWY-5898, PWY-5899, and PWY-5837) (Fig. 5C).

## Discussion

The growing interest in understanding the involvement of the microbiome in the pathogenesis of MAFLD has increased the number of publications focusing on this fact. Many intestinal bacterial populations have been identified in different studies as specific to be part of the microbial pattern in MAFLD, and the directions in which these may be modified in patients with the disease. However, our study assessed gut microbiota composition and functionality in a cohort of patients with morbid obesity and an accurate diagnosis of MAFLD by liver biopsy. Our data corroborated a significant distinguishing faecal microbiota composition in individuals with morbid obesity and MAFLD compared with those without MAFLD.

Nevertheless, individuals with morbid obesity and MAFLD (diagnosed by liver biopsy) showed no significant differences in the adjusted analyses for both alpha and beta diversity compared with those patients with morbid obesity without MAFLD. While other authors [22, 23] recently found a decrease in bacterial diversity in participants with MAFLD. The patient groups in the different studies differed according to MAFLD status (steatosis, fibrosis, NASH), age, and sex, among other aspects. In addition, while most of these previous studies relied mainly on imaging techniques, such as ultrasound, as a diagnostic method, our study used liver biopsy to diagnose MAFLD.

Likewise, we observed a significantly higher abundance of *Enterobacteriaceae* family and genera *Acidaminococcus* and *Megasphaera* in patients with MAFLD (steatosis plus NASH) than in the control group, and a significantly lower abundance of families *Eggerthellaceae* and *Ruminococcaceae*. These results on gut microbiota composition are in agreement with the literature, in which patients with MAFLD also showed a higher abundance of the *Enterobacteriaceae* family [24–26], an increase in *Megasphaera* genus [26], an expansion of gram-negative bacteria [23] such as *Acidaminococcus* genus, or a decrease in the abundance of *Ruminococcaceae* [23, 25, 27, 28], while another author found an increase in *Ruminococcus* genus [29].

Furthermore, our study found that specific OTUs were related to basic histological features in MAFLD biopsies. Consistent with the literature, an enrichment in *Enterobacteriaceae* [24–26, 30] and a decrease in *Ruminococcus* [23, 27, 28] was observed in fibrosis, steatosis, and necroinflammation, while an enrichment in *Escherichia* [24, 31] was associated with steatosis and necroinflammation. However, a higher abundance of *Ruminococcus* was independently associated with fibrosis [32]. On the other hand, enrichment in *Escherichia-Shigella* [25] and *Streptococcus* [26, 30] was associated with fibrosis, whereas a decrease in *Faecalibacterium* [28, 33] was observed in steatosis and enrichment in *Proteobacteria* with necroinflammation [24].

Overgrowth *Escherichia* and *Enterobacteriaceae* populations influences an increase in intestinal permeability and portal LPS levels, which could cause inflammation activation and contribute to liver injury [34]. Under normal conditions, alcohol is constantly produced in the human body, and the gut microbiota is the primary source of endogenous alcohol [35]. *Enterobacteriaceae* and *Escherichia* have been associated with overproduction of endogenous ethanol, which is a potential mechanism involved in the development and progression of liver injury [36, 37]. Under anaerobic conditions, *Enterobacteriaceae* takes the mixed-acid fermentation pathway, the major product of which is ethanol [38]. Specifically, ethanol, a microbial metabolite derived from saccharolytic fermentation by ethanol-producing bacteria, may be implicated in the progression of MAFLD through direct toxic effects on hepatic cells and alterations in intestinal barrier function (increased endotoxemia) [39]. Ethanol exposure can induce lipid deposition and exacerbate hepatic steatosis as well as hepatic inflammation and fibrosis. In addition, ethanol and its metabolites increase intestinal permeability and impaired the gut barrier [40, 41]. Meijnikman et al. [36] observed higher portal vein ethanol concentrations in patients with MAFLD than in those without steatosis, and these levels were higher with disease progression. In addition, endogenous ethanol production was abolished after antibiotic treatment, suggesting that microbial ethanol could be involved in MAFLD pathogenesis.

A decrease in *Ruminococcaceae* and *Faecalibacterium* was observed in patients with MAFLD. These bacteria are SCFA-producing bacteria produced by fermentation of soluble dietary fibre [42]. SCFAs are well known for their health-promoting functions, including energy supply to the colonic epithelium, modulation of colonic pH, maintenance of host immune homeostasis, and inhibition of proinflammatory functions [43]. Lower SCFA levels in the intestine potentially deteriorate intestinal integrity and increase intestinal inflammation and permeability, which are implicated in the MAFLD pathogenic [44].

In the bacterial communities of our cohort of patients with morbid obesity and liver injury, there was a significant reduction in OTUs belonging to the order *Clostridiales*, such as *Ruminococcaceae* and *Faecalibacterium*, which are involved in the pathogenesis of chronic liver disease [45].

In this study, we also characterized possible microbiome metabolite mechanisms for the development of MAFLD. We revealed enrichment of the super pathway of menaquinol-7 biosynthesis (K2 vitamin) (PWY-5840, PWY-5845, PWY-5850, PWY-5860, PWY-5861, PWY5862, PWY-5897, and PWY-5899) when comparing differential abundance between steatosis plus NASH group and control group. Menaquinones (K2 vitamins) are essential for humans and are usually supplemented by nutrient sources and gut bacteria such as *Escherichia coli, Bacteroides* species, and gram-positive bacteria [46, 47]. Previous studies have suggested a key role for vitamin K2 in recovering liver function in patients with liver cirrhosis by oval cell proliferation and liver regeneration in an animal model with rats [48]. Other properties of vitamin K2 include anti-inflammatory effects, such as suppressing the nuclear factor κB (NF-κB) signalling pathway [49] and promoting cancer cell apoptosis to suppress growth and differentiation in hepatocellular cancer [50]. Metagenomic analysis of the gut microbiome revealed enrichment of the menaquinone pathway in diabetes mellitus [51]. In our cohort of patients, the findings related to vitamin K2 production were centred on those who had steatosis plus NASH; therefore, they do not correspond to advanced stages of cirrhosis. More studies should be conducted focusing on the role of vitamin K2 in the evolution of the stages of liver disease and tissue regeneration.

It has been described that microbial products derived from saccharolytic and proteolytic fermentation can affect the gut-liver axis through multiple mechanisms, thus contributing to the pathogenesis of MAFLD. This fermentation mainly yields harmful products such as ethanol, ammonia, phenols, and branched-chain fatty acids, which might be negative for metabolic health [52]. Consistent with these studies, our findings showed increased metabolic pathways associated with enrichment in sugar acid degradation (GALACTARDEG-PWY and GLUCARGALACTSUPER-PWY) and amine and polyamine (ORNDEG-PWY and PWY-6071) in individuals with steatosis plus NASH compared with the control group.

Likewise, the gut microbiota can also produce important metabolites, such as succinate and SCFAs, through the fermentation of indigestible carbohydrates [52]. Our data showed an enrichment in pathways related to proteinogenic amino acid degradation, such as l-arginine and l-threonine degradation and succinate production (ARGDEG-PWY, ORNARGDEG-PWY, and THREOCAT-PWY) in the steatosis plus NASH group compared to control group. Succinate is an intermediate in the microbial synthesis of propionate. An increased abundance of succinate-producing bacteria and a decreased abundance of succinate-consuming bacteria have been associated with obesity and metabolic diseases such as impaired glucose homeostasis [53]. Therefore, succinate can stimulate the activation of hepatic stellate cells to produce extracellular matrix proteins, resulting in the progression of MAFLD to fibrosis and even cirrhosis [54].

## Conclusion

Our findings showed that patients with morbid obesity, steatosis, plus NASH (diagnosed by liver biopsies) were characterized by an altered microbial pattern with an increase in *Enterobacteriaceae* family, an ethanol-producing bacteria, and the depletion of *Ruminococcaceae* family, a health-promoting bacteria: SCFA-producing bacteria. MAFLD was also associated with enrichment in pathways related to proteinogenic amino acid degradation and succinate production, biosynthesis of menaquinol-7 (K2 vitamin), and saccharolytic and proteolytic fermentation. These metabolic pathways mainly produce harmful products, such as ethanol or succinate, resulting in possible mechanisms for the pathogenesis and progression of MAFLD. In addition, K2 vitamin biosynthesis, a molecule with anti-inflammatory and antitumor effects, is related to steatosis plus NASH (but not advanced stages).

Thus, we established a link between the altered microbiota patterns and histological injury in the liver. Gut microbiota analysis and their metabolic pathways could add information to the classical predictors of MAFLD severity and suggest novel metabolic targets.

### Supplementary Information

Below is the link to the electronic supplementary material.

Supplementary file1 Alpha-diversity gut microbiota in patients classified according to the presence of fibrosis, steatosis and necroinflammatory activity (PNG 67 KB)

Supplementary file2 Venn diagram showing enriched taxa in hepatic alterations (JPG 45 KB)

Supplementary file3 Relative abundance of the most representative altered taxa according to the grade of pathological feature liver disease (TIF 843 KB)

## Abbreviations

BMI: Body mass index
CCEIBA: Biomedical Research Ethic Coordinator Committee of Andalucía
HDL-c: High-density lipoprotein cholesterol
HOMA-IR: Homeostasis model assessment of insulin resistance
LDA: Linear discriminant analysis
LDL-c: Low-density lipoprotein cholesterol
LPS: Lipopolysaccharides
MAFLD: Metabolic-associated fatty liver disease
NAFLD: Non-alcoholic fatty liver disease
NASH: Non-alcoholic steatohepatitis
PICRUSt2: Phylogenetic investigation of communities by reconstruction of unobserved states
SCFA: Short chain fatty acids
SD: Standard deviation
TMA: Trimethylamine
