# Supplementary figures and images for: Non-alcoholic fatty liver disease in patients with morbid obesity: the gut microbiota axis as a potential pathophysiology mechanism
Source: J Gastroenterol. 2024 Jan 24;59(4):329–41. doi: 10.1007/s00535-023-02075-7 (PMC10959783; doi:10.1007/s00535-023-02075-7)

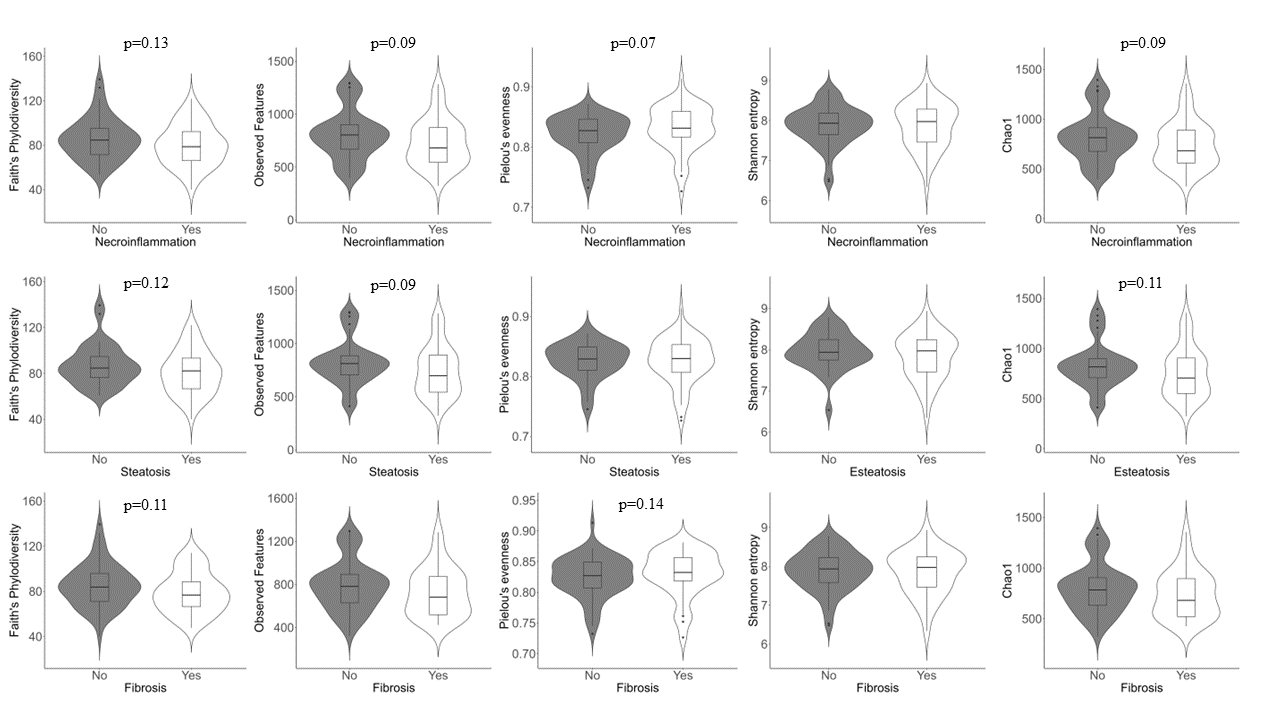

Supplement: Supplementary file 1 — Supplementary file1 Alpha-diversity gut microbiota in patients classified according to the presence of fibrosis, steatosis and necroinflammatory activity (PNG 67 KB) [file 535_2023_2075_MOESM1_ESM.png]

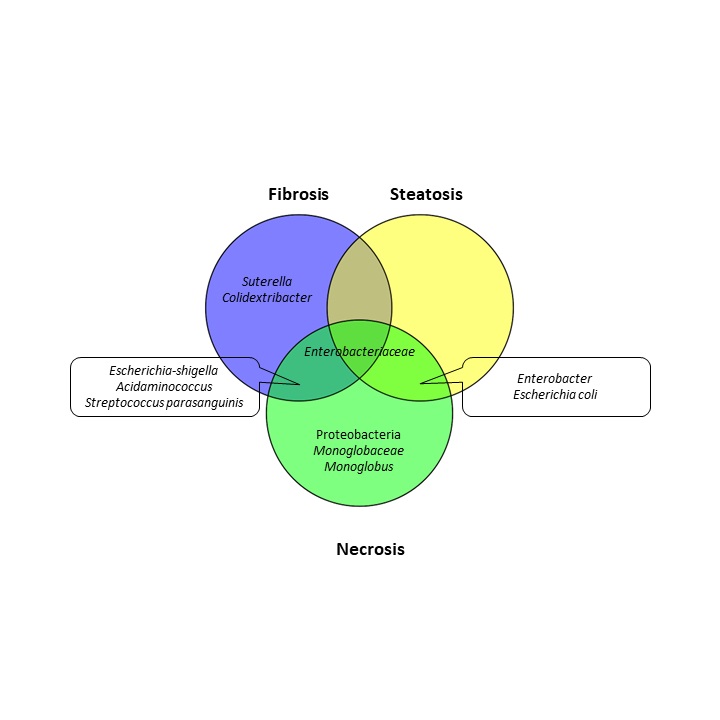

Supplement: Supplementary file 2 — Supplementary file2 Venn diagram showing enriched taxa in hepatic alterations (JPG 45 KB) [file 535_2023_2075_MOESM2_ESM.jpg]

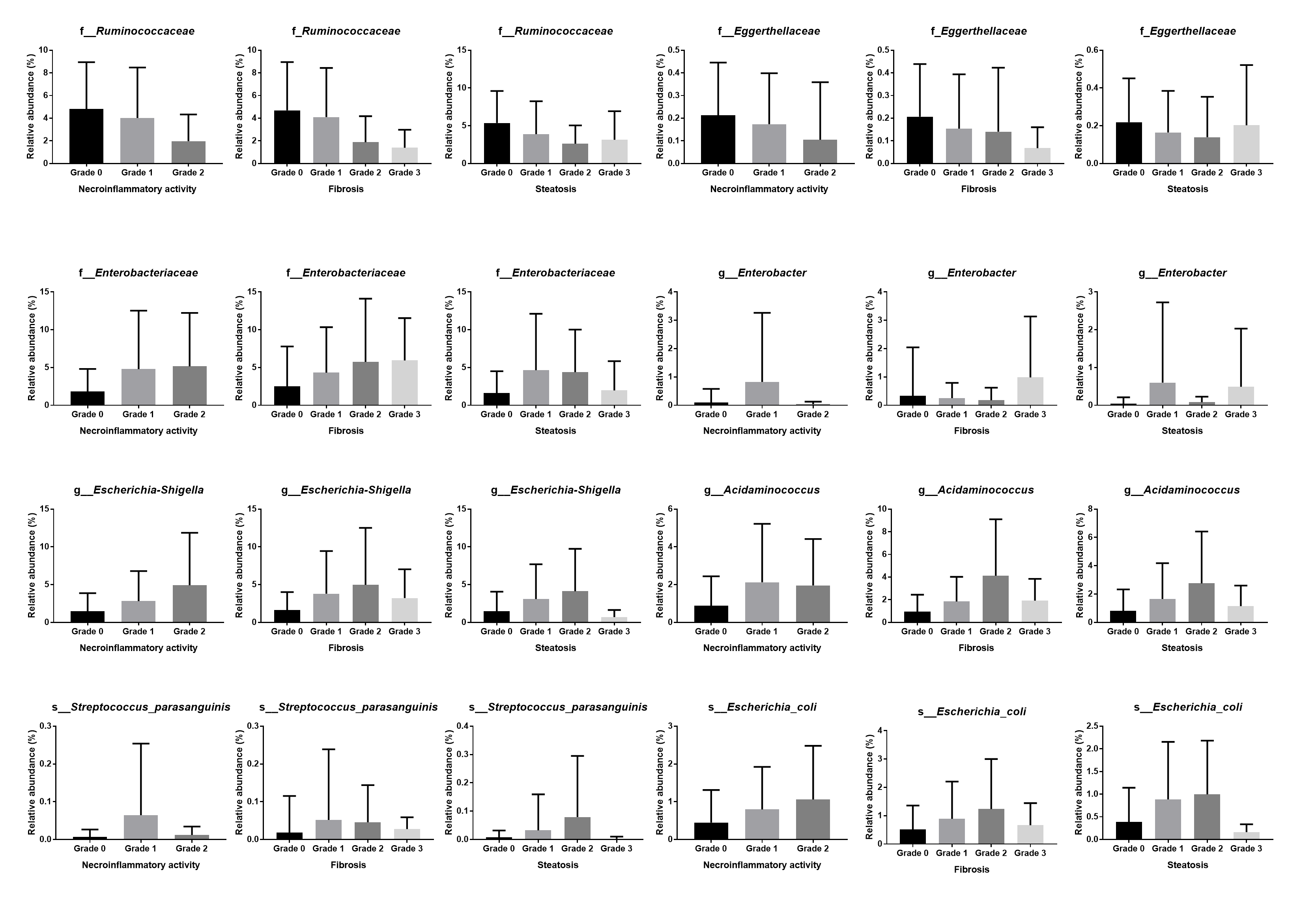

Supplement: Supplementary file 3 — Supplementary file3 Relative abundance of the most representative altered taxa according to the grade of pathological feature liver disease (TIF 843 KB) [file 535_2023_2075_MOESM3_ESM.tif]
